# The Synergistic Properties and Gas Sensing Performance of Functionalized Graphene-Based Sensors

**DOI:** 10.3390/ma15041326

**Published:** 2022-02-11

**Authors:** Zandile Dennis Leve, Emmanuel Iheanyichukwu Iwuoha, Natasha Ross

**Affiliations:** SensorLab, Chemistry Department, University of the Western Cape, Cape Town 7535, South Africa; 3135207@myuwc.ac.za (Z.D.L.); eiwuoha@uwc.ac.za (E.I.I.)

**Keywords:** graphene, graphene oxide, reduced graphene oxide, surface functionalization, gas sensor, metal oxide nanocomposites, gas sensing mechanism

## Abstract

The detection of toxic gases has long been a priority in industrial manufacturing, environmental monitoring, medical diagnosis, and national defense. The importance of gas sensing is not only of high benefit to such industries but also to the daily lives of people. Graphene-based gas sensors have elicited a lot of interest recently, due to the excellent physical properties of graphene and its derivatives, such as graphene oxide (GO) and reduced graphene oxide (rGO). Graphene oxide and rGO have been shown to offer large surface areas that extend their active sites for adsorbing gas molecules, thereby improving the sensitivity of the sensor. There are several literature reports on the promising functionalization of GO and rGO surfaces with metal oxide, for enhanced performance with regard to selectivity and sensitivity in gas sensing. These synthetic and functionalization methods provide the ideal combination/s required for enhanced gas sensors. In this review, the functionalization of graphene, synthesis of heterostructured nanohybrids, and the assessment of their collaborative performance towards gas-sensing applications are discussed.

## 1. Introduction

The globe has been faced with a burden of diseases linked to air pollution exposure which has had a massive toll on human health. The effects caused by exposure to air pollution have been estimated to cause millions of deaths and yearly losses of a healthy lifestyle. This burden has been reported to be on a par with other major global health risks, namely, unhealthy diets and tobacco smoking. Air pollutants are attributable as the single main environmental threat to the human health [1]. Air pollutants may be either released into the atmosphere, which may then be referred to as primary air pollutants, or formed within the atmosphere as secondary air pollutants. Primary air pollutants are composed of sulfur dioxide (SO_2_), oxides of nitrogen, carbon monoxide (CO), volatile organic compounds (VOCs), and carbonaceous and non-carbonaceous primary particles. Secondary air pollutants are formed from chemical reactions of primary air pollutants, which may often involve natural environmental components such as oxygen and water. These include ozone (O_3_), oxides of nitrogen, and secondary particulate matter (PM) [2].

Air pollution exposure is said to be largely determined by the concentration of air pollutants disposed in the environment to which people are exposed and the amount of time spent in that environment [2]. The World Health Organization (WHO) has, since 1987, periodically issued air quality guidelines (AQGs) based on health to better assist governments and civil society to reduce exposure to air pollution and its adverse effects. In 2005, WHO published AQGs for PM, O_3_, NO_2_, and SO_2_ [1]. Carbon monoxide was assessed in 2000 and later, in 2010, as an indoor pollutant [3]. Table 1 shows the WHO AQGs set for health protection based on air pollutant concentrations and average times for short-term and long-term exposures. These were later updated, and the latest data showed that PM_10_ had a 15 µg/m^3^ annual mean and a 45 µg/m^3^ 24-h mean; PM_2.5_, a 5 µg/m^3^ annual mean and 15 µg/m^3^ 24-h mean; O_3_, a 100 µg/m^3^ 8-h daily maximum and 60 µg/m^3^ 8-h mean on a six-month basis; NO_2_, a 10 µg/m^3^ annual mean and 25 µg/m^3^ 24-h mean; SO_2_, a 40 µg/m^3^ 24-h mean [3].

The generation of toxic gases including nitrogen oxides (NOx), sulfur oxides (SOx), ammonia (NH_3_), and CO has been stated as a major hazard to environmental security and individual health protection [5]. The detection of NO_2_ has generated substantial attention as it is not only harmful for the respiratory system but also causes acid rain formation [6]. Hydrogen sulfide (H_2_S), which is also a toxic gas produced from the process of oil and natural gas production, is highly dangerous for the human body with reported health effects following exposure including death and respiratory, ocular, neurological, cardiovascular, metabolic, and reproductive effects [7]. Ammonia (NH_3_) is an irritant and corrosive gas, to the extent such that its low concentrations in air or liquid can lead to severe irritations and coughing in the case of skin or eye contact [6]. Carbon monoxide (CO) is also highly toxic to humans, amongst various gases, as it is an odorless, colorless, and tasteless gas which appears to be slightly denser than air, therefore making it difficult to recognize in a normal way [8]. Therefore, the detection of toxic gases and harmful chemical vapors within a limited time is of the utmost importance.

The importance of gas sensors has long been apparent in different aspects of certain fields since the first invention by Davy in 1815 [9,10] and a commercial catalytic combustion gas detector by Johnson in 1926 [11], for which first substantial studies began in the early 1970s and later rapidly expanded since 2002. Hence, simple, and accurate detection of toxic gas has become vital in our everyday lives, not only to industries but to all people [7]. Techniques such as optical, acoustic and gas chromatography, chemiluminescence ion chromatography, and spectrophotometry have been utilized for the detection toxic gases [12,13]. However, the methods mentioned are not cost-effective, complex, and are not suitable for implementation for widespread, continuous monitoring in ambient conditions. As a result, electrochemical sensing is the most widely used method for detecting dangerous gases. Electrochemical detection has several advantages over other approaches, including strong selectivity and repeatability, ppm level detection with high precision, low energy linear output with high resolution, and lower cost. In recent years, electrochemical sensors made of diverse functional materials have been the focus of harmful gas detection research [7].

### Principles of Graphene-Based Gas Sensors

Graphene is described as a flat one-atom thick monolayer of sp^2^-hybridized carbon atoms that are tightly stacked into a two-dimensional honeycomb lattice [14]. Its semimetal nature allows charge carriers to behave like Dirac fermions which results in extraordinary effects such as improved intrinsic mobility of up to ~200,000 cm^2^·V^−1^·s^−1^, with unique properties such as a higher thermal conductivity of ~5000 W·m^−1^·k^−1^, high mechanical stiffness of ~1060 GPa, an excellent optical transmittance of ~97.7%, and a large specific surface area of 2630 m^2^·g^−1^ [15]. Graphene is a basic building block for graphitic materials of all other dimensionalities [16].

Graphene oxide (GO) is an oxide form of graphene that is covered by a high density of oxygen functional groups such as hydroxyl, epoxy, and carboxyl on its basal plane and carboxyl on its edges, making it easy to suspend in water and other polar media [15]. Its carbon atoms are partially sp^3^-hybridized and they can move above or below the graphene plane [17]. The ability of GO conduction depends on the degree of oxidation in the compound and the synthetic route proposed. Graphene-like sheets are produced by reduction of GO in which the oxygen functional groups are removed whilst recovering the π-conjugated network, which is the most fascinating property of GO [18].

A material related to GO is reduced graphene oxide (rGO), which possesses sheets that are regarded as chemically derived graphene [18]. Measurements of elemental analysis (atomic C/O ratio, ~10) for rGO performed by combustion reveal that a significant amount of oxygen exists in the structure, which indicates that rGO is not the same as pristine graphene (Park and Ruoff, 2009). Additionally, its conductance decreases by a magnitude of three orders when cooled to lower temperatures, which makes it exhibit non-metallic behavior whilst it is nearly metallic [19].

Graphene has emerged as a possible contender for sensing applications, among other things. Experimental and theoretical research has reported on the demonstrated monolayer graphene as a promising candidate to detect a variety of molecules, including gases, due to its appealing advantages [20]. Graphene oxide is a popular precursor of graphene because of its high water solubility, ease of functionalization, and simple processing [21]. In an as-oxidized state, GO has poor conductivity [22] as it is rendered too electrically insulating as a conductance-based sensor due to the disruption of the π-conjugated system by oxygen moieties [20,23]. Chemical reduction partially restores the conductivity by the removal of oxygen which then recovers the aromatic carbon double bonds. Yet, this still does not repair to pure graphene, as some oxygen groups remain in the network [24]. The rGO has intermediate conductivity and defect sites which make it attractive for sensor application [24].

Functional materials have been reported to be used in chemiresistive gas sensors. Volanti et al. [25] report on the development of CuO-based nanostructured chemiresistive gas sensors with different morphologies, which were exposed to oxidizing and reducing gases in the same test chamber over a range of temperatures and gas concentrations that were measured simultaneously [25]. However, chemiresistive gas sensors have been reported to have drawbacks such as a lack of selectivity, flexibility, high power consumption, safety risks, and a high operating temperature [26]. Nanostructured materials, such as conductive polymers (CPs), have been studied extensively around the world because they have unique and intriguing properties such as ease of synthesis, structural diversity, environmental stability, low cost, flexibility, and a sensitive response to chemical molecules at room temperature [27]. Conducting polymers possess a strong potential for producing enhanced sensor performance in comparison to their bulky opposite [28]. However, they lack stability at the nanoscale attributed to the nature of covalent bonds, also resulting in an unstable nanostructure [26]. Due to this factor, progress in the synthesis of CPs has been reported to be relatively slow with limited research when compared to inorganic nanomaterials [29].

Metal oxides have been used as a sensing material in low-cost sensors. Due to their good sensing capability, fast response, and recovery, gas sensing devices based on metal oxide sensors have been thoroughly investigated. These sensors do, however, have an operational limitation due to their failure to work at temperatures much higher than room temperature. Complex circuitry and high power consumption are required for operation or optimal response at higher temperatures [6]. There are some methods that have been successfully employed to enhance the selectivity of metal oxide sensors such as the optimization of temperature, bulk/surface doping, and the use of molecular filters [30]. The exceptional properties of graphene, GO, and rGO pose them as highly useful materials for various applications by the functionalization/doping of surfaces in a variety of ways and hence are widely investigated by researchers [18,22]. In this work, the synthesis and fabrication of GO/rGO/nanoscale metal oxide nanocomposites and assessing their performance for gas/vapor-sensing applications are reviewed.

## 2. Synthesis of Graphene-Based Inorganic Nanostructured Composites

**Figure 1:**
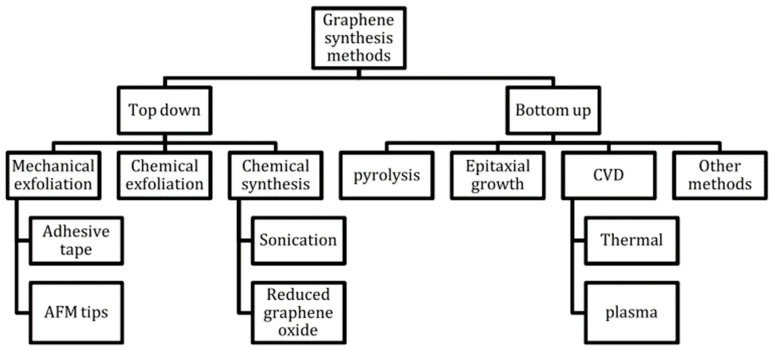
Summary of the graphene synthesis methods [14].

Graphene has been synthesized using various methods as shown in Figure 1. These include mechanical exfoliation [31], chemical exfoliation [32], epitaxial growth on silicon carbide (SiC) [33,34], and chemical vapor deposition (CVD) [35]. Mechanical exfoliation is described as a simple peeling process involving a commercially available highly oriented pyrolytic graphite (HOPG) sheet that is dry etched in oxygen. It may produce graphene with exceptional properties, but it is limited by its low production which may not be sufficient for specific applications [31]. Chemical oxidation of graphite and its subsequent exfoliation lead to a greater amount of graphite monolayer, and the chemical treatment inevitably results in structural defects. The graphene can be used in industrial applications such as paint additives or composites [32,36].

**Figure 2:**
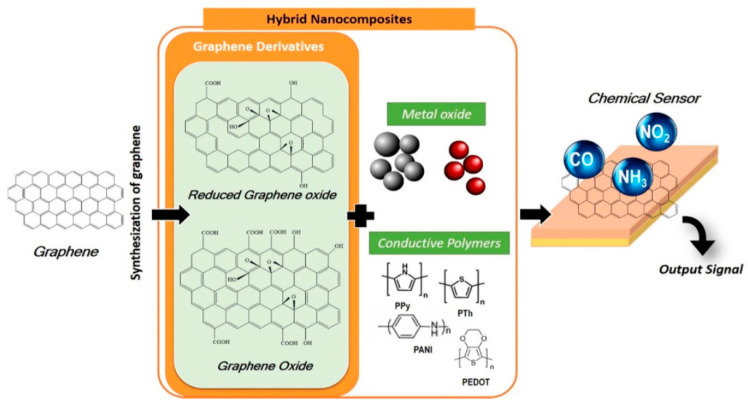
Schematic of graphene hybrid nanocomposites’ fabrication into chemical sensors [39].

In thermal decomposition of SiC, the compound is heated under an ultrahigh vacuum (UHV) and the Si atoms sublimate from the substrate. The formed few-layer graphene (FLG) typically needs a short period of time to anneal at a temperature of 1200 °C. The graphene layers can be grown directly on a semiconducting substrate, but production is still not producible [37]. In CVD, gas species are placed in a reactor and passed through the hot zone in which hydrocarbon precursors are decomposed into carbon radicals at the surface of a metal substrate, and, thereafter, a single-layer and few-layer graphene are formed [35]. The method produces a large area and high-quality graphene, and it is also inexpensive [38]. Chemically derived graphene is achieved by synthesis of GO and its subsequent reduction into rGO [16]. In a typical procedure, graphite undergoes chemical oxidation into GO using a modified Hummers’ method. The abundance of functional groups in GO results in hydrophilic behavior which is strongly dependent on the level of oxidation [39]. Reduction of GO follows thereafter to form rGO via several methods such as thermal or chemical reduction and electrochemical reduction which produce mass production of rGO [18]. Summary of the synthesis of graphene with different routes is shown in Figure 2.

Graphene-semiconductor nanocomposites could open new possibilities for graphene-based catalytic and photocatalytic reactions [40]. To improve the properties of graphene-based composites, metal nanoparticles, metal oxides, and other inorganic compounds have been made into a structure with graphene [41]. Metals such as Au [41], Ag [42], Ni [43], Cu [44], Ru [41], Rh [45], and Co [46,47], along with metal oxides such as TiO_2_ [48], ZnO [49], MnO_2_ [50], Co_3_O_4_ [51], NiO [52], and Fe_3_O_4_ [53], and metal organic framework ZIF-8 [54] were among the materials used. There have also been graphene/MoS_2_ and graphene/silica nanocomposites created both of which have fascinating electrical characteristics [55]. Coulombic charge transfer between noble metal NPs and graphene has been demonstrated during their interaction [56,57]. Through excited-state electron transport, graphene oxide interacts with NPs of semiconducting oxides [58,59]. Figure 2 shows a graphical representation of the creation of chemical sensors using graphene derivatives and metal NPs/metal oxides.

Inorganic nanostructures have been used to make graphene-based composites [42,49,51,60], and graphene has been employed as a novel and promising platform for the synthesis of graphene-based noble metal nanostructures [60,61]. Huge efforts have been undertaken in recent decades to create inorganic nanostructures with regulated shapes, size, crystallinity, and functionality [62,63]. Belts, tubes, rods, wires, particles, and polyhedrons are examples of inorganic components with a variety of morphologies [64]. By combining graphene and its derivatives with various types of functional materials in composites, it is possible to harness their desirable qualities [20,65,66,67,68,69]. The production of unique noble metal nanocrystals and their prospective applications in varied domains such as catalysis, electronics, sensing, and medicine have made significant development [70,71,72,73,74,75]. A novel class of material system for constructing unconventional inorganic electrical and optoelectronic devices is the hybrid heterostructure, which is made up of inorganic nanostructures grown directly atop graphene layers [15,76,77].

The high carrier mobility, radiative recombination rate, and long-term stability of inorganic semiconductor nanostructures enable the creation of high-performance optoelectronic and electrical devices [78,79,80]. Graphene–inorganic hybrid materials have been created in the last few years by inserting inorganic nanostructures between graphene sheets using the driving force of chemisorption interaction [81,82]. The methods for the synthesis of graphene-based inorganic hybrid materials can be divided into two categories: (i) graphene (oxides) assembly with generated inorganic nanostructures and (ii) graphene and inorganic nanostructure synthesis and assembly in one pot. The first method involves preparing inorganic nanostructures before mixing them with graphene or GO dispersion. The second technique, on the other hand, involves obtaining graphene and inorganic species in situ and then assembling them in a single-pot synthesis [83].

Alfano et al. [84] developed a sensitive material in which graphene was created by exfoliating graphite in its liquid phase, and then followed by microwave functionalization with ZnO. In comparison to equivalent devices made of bare graphene, chemiresistor devices made of hybrid materials showed that adding ZnO NPs to the graphene matrix can increase the sensing platform by increasing the sensing response and enhancing the selectivity. Wu et al. [85] created an inkjet-printed graphene-MOx-based sensor system that can be integrated onto tiny CMOS-compatible platforms to measure NH_3_ selectively and accurately. Inkjet printing was employed to deposit ZnO-graphene functional inks directly onto the interdigitated Au electrodes (IDEs) on the Si_3_N_4_ membrane substrate (5 m finger width and gap; 250 m diameter) of the CMOS HP during the sensor fabrication process. The process described allowed for the automated manufacture of many devices at the same time. Kim et al. [86] developed a method for producing graphene/SnO_2_ nanocomposites via explosive microwave synthesis for use in gas sensors. The fabrication technique was able to achieve rapid and large-scale production thanks to the fast surface chemical reactions enabled by microwave-generated plasmas, which could lead to the commercialization of semiconductor-type gas sensors by lowering production costs and improving sensing capabilities.

In comparison with graphene, GO presents advantages such as a low production cost, large-scale production, and easy processing [17,87]. A variety of materials have been created on GO or rGO nanosheets including inorganic nanostructures, organic crystals, polymers, metal organic frameworks (MOFs), biomaterials, and carbon nanotubes (CNTs) [60,61,88,89,90,91,92,93,94,95]. Kavinkumar and Manivannan [96] used chemical reduction with vitamin C in GO suspension to make AgNPs–GO composites with various AgNO_3_ concentrations. Following that, an AgNPs–GO-coated fiber optic gas sensor for NH_3_ detection was created. The composites were proven to have superior sensitivity and sensing performances to rGO. Jiang et al. [97] conducted a study in which nanoparticles and GO of SnO_2_/NiO were produced. The GO/SnO_2_/NiO materials were created using a hydrothermal growth composition in a neutral environment. A gas sensitivity test system was used to determine the composite’s sensitivity, ideal working temperature, and selectivity.

Graphene oxide is typically used as a precursor in the preparation of rGO (Yu et al., 2020; Dreyer et al., 2010). Li et al. [98] used a one-pot solution approach at room temperature to successfully manufacture CuO/rGO nanohybrids, in which the reduction of GO and the synthesis of CuO occurred simultaneously. The sheet-like CuO produced was found to be consistently mixed with rGO nanosheets [98]. Gu et al. [99] stated that they have effectively manufactured n-type In_2_O_3_–rGO nanocomposites using a simple hydrothermal technique, with great selectivity, high response, and a quick response/recovery time. The experimental results showed that the In_2_O_3_–rGO nanocomposite-based gas sensor had a good chance of being a good candidate for NO_2_ monitoring in the environment. Karthik et al. [100] used a spray pyrolysis technique combined with an annealing process to make rGO/TiO_2_ thin films. The entire sensing nature of the rGO/TiO_2_ sensor was allegedly owing to the design component of rGO, which reduced TiO_2_ nanoparticle accumulation and advanced porosity and conductivity.

Zhang et al. [101] developed an rGO/SnO_2_/Au sensor in which rGO/SnO_2_ nanocomposites were decorated with Au nanoparticles at high concentrations of a GO precursor, which was obtained by adding HAuCl_4_ and SnCl_2_ to the reaction system. Following that, hybrid nanomaterials were used as gas sensors, and they performed well. Wang et al. [102] successfully manufactured a gas sensor based on AgNPs–SnO_2_–rGO hybrids synthesized using the hydrothermal synthesis approach. When compared to SnO_2_–rGO hybrids, the gas-sensing results showed that adding AgNPs to the SnO_2_–rGO hybrids considerably improved the gas-sensing capability at room temperature. Ifterkhar Uddin et al. [103] used a simple chemical approach to construct a gas sensor based on a Ag-loaded ZnO–rGO hybrid to improve gas-sensing performance at low working temperatures. The as-synthesized sensing material was characterized and found to have a homogeneously dispersed and tightly adhered Ag–ZnO mixer on the surface of the reduced graphene oxide. The gas sensor performed best at 150 °C, with three wt% Ag-loaded ZnO–Gr exhibiting improved sensing properties as compared to individual equivalents.

## 3. Surface Functionalization of Graphene/GO/rGO with Metal Oxide Nanocomposites towards Gas Sensing

In general, different gases possess molecules with electron-withdrawing or -donating abilities that can adsorb onto the surface of graphene and thereby alter its conductivity. The sensing platform of this nature has intrinsically high sensitivity. This can be attributed to the conical band structure of graphene that ensures significant conductivity changes. Nonetheless, selectivity is an issue for a sensitive chemiresistor where many gases can result in large sensing responses. Therefore, functionalization of graphene surfaces has been proposed as a solution to this issue [104]. Chemical functionalization is a powerful tool for modifying structure and specific characteristics of graphene. This can be done via non-covalent as well as covalent routes according to the operation between the ligands and the sp^2^ carbonaceous lattice [105]. In the former, the extended π-electron delocalization of the graphene sheet remains intact, whereas the latter takes place via the formation of covalent bonds between the graphene and different organic (inorganic) functional groups [105]. Combining graphene with newly added groups in the form of covalent bonds to improve and increase its performance is known as covalent bond functionalization [17,106,107,108]. By interacting of hydrogen bonds and the electrostatic forces between graphene and functional molecules, non-covalent bond functionalization of graphene or graphene oxide results in the formation of a composite material with a specific function, the greatest advantage of which is maintaining the bulk structure and excellent properties of graphene or graphene oxide, as well as improving the dispersibility and stability of graphene or graphene oxide [109,110].

Graphene on its own does not exhibit good sensing properties; however, derivatives have shown exceptional sensing ability due to better optical, mechanical, electrical, and chemical properties [99,111,112]. To combine different elements into graphene, element doping modification typically uses annealing heat treatment, ion bombardment, arc discharge, and other methods, resulting in the substitution of defects and vacancy defects in graphene while maintaining the intrinsic two-dimensional structure of graphene [113,114,115]. Generally, when the electrical conductivity and large surface of graphene are required, non-covalent modification methods are typically preferred. Likewise, when the stability and the strong mechanical properties of modified graphene are expected, covalent methods are ideal. Graphene sheets can be uniformly disseminated in aqueous and/or organic (inorganic) fluids by selectively adding functions to their surfaces [108,110,113,116,117]. Graphene oxide lacks chemical reactivity, which can be attributed to its homogeneity and highly delocalized electronic structure. In typical occurrence, chemical reactions are traced at locations that exhibit weak or labile bonds, highly localized orbitals, dangling bonds, or localized charges [117]. All these cannot be found in graphene, whilst in its honeycomb lattice structure, each sp^2^ atom of carbon is characterized by a 3-fold symmetric electronic hybridization where the p-orbitals extend out of the atomic plane [118]. In this manner, a self-passivating and highly delocalized network is formed [113]. Disruption of this chemical structure is not only thermodynamically unfavorable, it also requires the formation of high-energy radicals on adjacent carbons which are difficult to support [117].

The oxides which are functionalized with various oxygen groups produce GO and rGO, which provide more adsorption sites for gases and so improve the sensitivity of the film. The presence of oxygen groups in GO renders it an insulating material [119] and since it is difficult to control the content of these groups during oxidation, GO is not an appropriate gas-sensing material. Hence, GO is reduced into rGO [120], and the generation of some oxygen functional groups that remain following reduction, coupled with some defects and vacancies, prove beneficial for gas adsorption [121]. These lead to electron transfer from rGO to the oxygen functional groups located at the surface of rGO. The holes are the main charge carriers and thus make rGO act as a p-type semiconductor [122]. Graphene or its derivatives provides faster carrier transport through barrier by opening of the sizable energy gap due to quantum confinement [123]. In this way, they can be exploited as catalytic active centers for covalent/non-covalent modification design, depending on the needs of particular application domains [17,32]. Furthermore, the presence of oxygen-containing groups broadens the graphene oxide interlayer gap. Small molecules or polymer intercalations can be used to functionalize it [124]. As a result, increasing the applications of graphene and graphene oxide requires functional modification [17]. Kumar and Kaur [125] described how thermal annealing reduced the electrical gas sensing of graphene oxide. The number of graphitic domains, as well as the specific surface area and pore volume, were found to increase the sample’s gas-sensing ability when exposed to SO_2_.

Chemical functionalization of graphene using synthetic chemistry methods allows for the creation of p- and n-doped graphene by selecting electron-donating or electron-withdrawing complexes that are covalently bound to the graphene carbon network. The doping concentration could often control the electrical characteristics [126]. The bandgap would open at the Fermi level of graphene as a result of successful doping, and graphene’s ‘metallic’ character would be transformed to a ‘semiconductor’ one [106,127,128,129]. For n-type (p-type) doping, electrons must be released into (or extracted from) the graphene layer, which is commonly accomplished by adsorbing atoms and/or molecules on its surface, i.e., surface transfer doping [126,128,130,131,132]. P-type doping for graphene is a lot more difficult [133]. For strong dimer bonds, many elements with a high electronegativity, such as nitrogen, oxygen, or fluorine, are used. On the graphene surface, they are unlikely to form a stable layer.

To generate p-type doping in graphene, several chemicals such as NO_2_, H_2_O, NH_3_, or charge transfer complexes have been utilized. However, NO_2_, H_2_O, and NH_3_ are highly reactive compounds that should not be used in electronic materials [128,133,134,135,136]. The heavier elements, which are less reactive than oxygen or fluorine, offer feasible options. Bismuth and antimony, while having a lower electron affinity than atomic carbon, are able to pull electrons from the graphene sheet, which is not immediately apparent. The functionalization of graphene and graphene oxide is achieved by altering their intrinsic structure further [137]. The correct functionalization of graphene and graphene oxide prevents agglomeration and protects their inherent properties during the reduction process. The functional modification of graphene and graphene oxide preserves their remarkable properties while also introducing additional functional groups to offer them new properties [128].

Hybridization with metal oxide nanostructures improves graphene-based sensors even more [123] by offering a higher surface-to-volume ratio with good adsorption of gas molecules on the sensor surface at numerous active sites [6,138]. This causes variations in the carrier concentration of graphene-based metal oxide composite film and, thus, the resistance of the film. The change in resistivity allows sensors to identify the target gas as an electron donor or acceptor [99,103,111,112]. Furthermore, graphene’s properties prevent metal oxide agglomeration, whilst in turn the metal oxides prevent graphene fossilization [6]. There have been several reports on the functionalization of graphene for gas-sensing applications throughout literature [139]. Graphene-based gas sensors can overcome the limitations on which traditional sensors fall short, such as sensitivity and selectivity coupled with power consumption, temperature-dependency that is significantly large, and safety issues [140]. Wicaksono et al. [141] exhibited the gas-sensing characteristics of graphene–TiO_2_/TiSiO-coated fabrics towards various gases. Martinez-Orozco et al. [142] presented a study on the preparation of a hydrogen-gas sensor, synthesized by the microwave method, based on Pd nanoparticles which were dispersed and anchored on graphene layers. The methodology utilized allows for the synthesis of functional Pd–graphene nanostructures.

A wet chemical route through Hummers’ method for the synthesis of functionalized rGO was achieved by Panda et al. [8], for which the sensing properties (~71% sensitivity against 30 ppm CO) were used to detect low-concentration CO at room temperature and atmospheric pressure under ambient humidity. Muda et al. [143] also reported on the fabrication of a gas sensor based on a vacuum method to achieve a homogenous and uniform thin film of multi-layer rGO on a plastic substrate. The fabricated sensor was used to detect NO_2_, and a sensitivity of ~25% over 50 ppm gas concentration was reported. However, the recovery was slow as it took time to fully recover to its baseline resistance before the exposure timescales were tested.

In regard to the response time, Jia and Wang [5] reported on a novel NO_2_ gas sensor where rGO adsorbs NO_2_ gas molecules as well as transports electrical signals, and the AgNPs act as catalysts to enhance the sensing response. Kang et al. [144] designed an rGO gas sensor functionalized with SnO_2_ nanoclusters in order to improve the recovery performance. The sensor was used to measure NO_2_ under UV illumination, and it was discovered that the rGO device that was functional near the percolation threshold had the best recovery and then the best sensitivity in subsequent cycles. The rGO–metal-oxide semiconductor nanocomposites, however, were reported to be not suitable to detect other gases, such as H_2_, CO, and C_2_H_5_OH, from a power consumption point of view and may not be favorable due to their operation at high temperatures [141].

## 4. Morphological Influence of Graphene-Based Metal Oxide Nanocomposite in a Gas Sensing Mechanism

Karthik et al. [100] studied the fabrication of rGO nanosheets functionalized with titanium dioxide (TiO_2_) towards CO_2_ gas. The gas-sensing mechanism involved CO reacting when it came into contact with ionized oxygen. As reaction products, CO_2_ and surplus electrons were to be emitted and the extra electrons contributed to the material’s increased conductivity. When compared to the air atmosphere, the nanocomposite material showed a decrease in resistance, which led to an increase in conductivity owing to the synergistic effect of GO and TiO_2_ nanoparticles. This was attributed to the higher charge-carrier density at the nanocomposite material surfaces due to adsorbed CO. When these two come together, the n-n intersection is detected, and the charge carriers are transferred from TiO_2_ to GO. Due to the tall depletion layer of TiO_2_, handle electrons in GO were rapidly incremented at the interface. The chemisorbed interaction between oxygen atoms from GO and gas atoms was mostly responsible for the change in resistance. The sensing nature of the rGO/TiO_2_ sensors was attributed to the design component of rGO, which reduces TiO_2_ nanoparticle accumulation and therefore advances porosity and conductivity.

**Figure 3:**
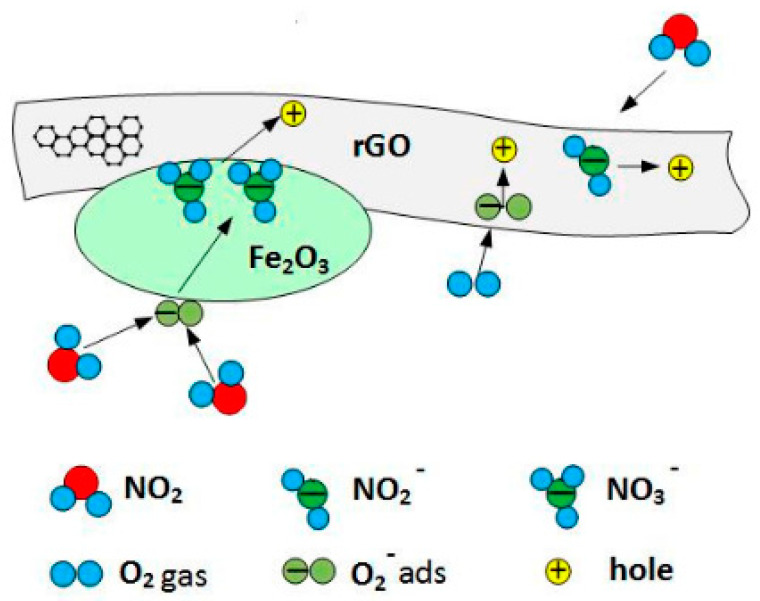
Interaction of NO_2_ gas with oxygen adsorbed on Fe_2_O_3_ surface can effectively increase the concentration of holes in rGO [145].

A technique used by Tadeusz Pisarkiewicz and co-workers [145] demonstrates Fe_2_O_3_ as an n-type semiconductor, but in the rGO/Fe_2_O_3_ hybrid structure it behaves similarly to p-type rGO. Both chemisorbed O_2_ and NO_2_ act as electron traps, decreasing the concentration of electrons, with a decrease of resistance (hole density increases). Pure Fe_2_O_3_ is nearly insensitive to NO_2_, but, within an rGO/Fe_2_O_3_ composite, NO_2_ reacts with O^2−^ adsorbed on the Fe_2_O_3_ surface, resulting in the formation of an intermediate NO_3_ complex. The unbalanced charges on the Fe_2_O_3_ surface are compensated by the transfer of additional electrons from rGO to Fe_2_O_3_, which results in additional holes in the rGO and increased conductivity, as shown in Figure 3. At the interface between rGO flakes and Fe_2_O_3_ grains, the p-n heterojunctions can be formed. The concentration of holes in the accumulation layer increases after interaction with NO_2_, leading to the increased conductivity of GO flakes in the presence of NO_2_ gas.

A tin oxide (SnO_2_)/rGO/polyaniline (PANI) sensor constructed by Zhang et al. [146] displayed superior H_2_S-gas-detection capabilities. The SnO_2_/rGO/PANI nanocomposites, with PANI, rGO, and SnO_2_, were tightly wrapped together to form a porous nanostructure. In the heterostructure, two different types of depletion layers were seen during the gas sensing process. The adsorption of oxygen (O) species at the surface of SnO_2_ was attributed to the first depletion layer, whereas the other depletion layer was linked to the SnO_2_ and PANI heterojunction. The performance of chemiresistive gas sensors has been shown to be influenced by the sensing properties of metal-oxide-based surface reactions between chemically adsorbed oxygen species [147]. The electron depletion layer was formed on the SnO_2_ surface by the chemisorption of oxygen species [146,148]. Adsorbed oxygen species (O^2^-(ads)) adhered to the SnO_2_ surface of the SnO_2_/rGO/PANI film [149]. As a result, a thicker electron depletion layer formed on the sensing film’s surface, causing the sensor to have a high resistance state in the air. A substantial number of electrons were liberated into the conduction band of metal oxide when the adsorbed oxygen species reacted with H_2_S. The sensor resistance was reduced when the thickness of the electron depletion layer was reduced [150]. The porous nanostructure of the SnO_2_/rGO/PANI heterojunction was shown to contribute significantly towards enhancing the H_2_S sensing properties [146].

To detect SO_2_ gas, Zhang et al. [151] used TiO_2_/rGO nanocomposite metal organic frameworks (MOFs) as sensor platforms. The sensing process involved SO_2_ molecules dissociating and adsorbing on the accessible apertures. O_2_ around the sensing material was surely adsorbed on the surface of the non-stoichiometric TiO_2_ and transformed to an O species by releasing a hole due to the gap of the oxygen atom [152]. When a significant number of holes on the p-type TiO_2_ surface were facilitated as the majority of carriers, the resistance of the MOFs TiO_2_ decreased. When the sensor was exposed to air, a portion of the oxygen molecules decomposed into O ions, releasing holes (h^+^) inside the Debye length. Upon exposure of the sensor in a SO_2_ atmosphere, the amount of O ions on the surface of the MOFs TiO_2_ and its positive-charge hole (h^+^) were reduced compared to when the sensor was in a dry gas, which resulted in a decrease in the majority carrier concentration, and thus the resistance of the TiO_2_ film climbed. When the sensor was exposed to SO_2_, O ions adsorbed on the surface of the MOFs TiO_2_ interacted with SO_2_ (reducing gas), resulting in the production of unstable SO_3_ via trapping holes in the surface of the MOFs TiO_2_. The number of positively charged carriers began to reduce, resulting in a decrease in the majority carrier concentration, causing the resistance of the MOFs TiO_2_/rGO film sensor to rapidly increase [153]. In the MOFs TiO_2_/rGO nanocomposite, p-type semiconductor activity was observed. The resistance of p-type nanomaterials was affected by the thickness of the hole accumulation layer (HAL), which resulted in poor resistance [151].

Revolved around the discussed works, graphene-based resistive gas sensors possess the advantages of rapid responsivity, outstanding sensitivity, excellent repeatability, and stability. Zhang et al. [146] compared the H_2_S sensing properties of SnO_2_, SnO_2_/PANI, SnO_2_/rGO, and in situ polymerized SnO_2_/rGO/PANI sensors at 25 °C, observing that the responses of the in situ polymerized SnO_2_/rGO/PANI sensor were about 3.18%, 8.34%, 24.07%, 32.16%, 44.91%, 60.11%, 76.25%, and 91.11% toward the corresponding H_2_S concentration of 50 ppb, 100 ppb, 200 ppb, 500 ppb, 1 ppm, 2 ppm, 5 ppm, and 10 ppm, indicating that the sensor can achieve a sub-ppm-level detection of 50 ppb H_2_S gas. The sensor also showed long-term stability where it was measured every day over a period of a month; the little change in response confirmed its good long-term stability. The rGO/TiO_2_ sensor outperformed the bare rGO and TiO_2_ sensors in terms of sensitivity, with the rGO/TiO_2_ sensor having a maximum sensitivity of 77% compared to 38% for the bare rGO and TiO_2_ sensors with over 1500 ppm H_2_S, respectively. Similar results were achieved for CO_2_ gas sensitivities, which were 42% and 92% for the bare TiO_2_ and rGO/TiO_2_ sensors, respectively, and these were also measured with a gas concentration of against 1500 ppm [100]. The MOF-derived rGO/TiO_2_ sensor demonstrated good reproducibility for SO_2_ sensing at 1, 3, and 5 ppm over a time span of 0 to 1600 s [151]. Hence, graphene-based gas sensors are considered to be among the most ideal for toxic gas detection. There are however still the concerns of poor selectivity and high operating temperatures. To reduce the working temperature of the resistive gas sensor, it is necessary to innovatively develop high-performance, low-temperature gas-sensing materials and further clarify their working mechanisms. Concomitantly, the causes of selective behavior are not completely understood to date [6]. At present, to advance gas-sensing performances, the best option is to optimize the gas-sensitive materials via doping, heterostructures, and composites. These methods can adjust the grain size, porosity, and specific surface area of the material, improve the electron transport characteristics, and increase the surface adsorption at the active sites, thereby improving the sensitivity and selectivity of the gas sensors.

## 5. Conclusions

A major cause of the rising environmental hazards is toxins in the atmosphere. The scientific community continually examines new sensing materials for environmental gases at a laboratory level (concentrations at ppm and ppb scales; absorption of target gas molecules at low and high ranges) which shows good performances. In this review, we focused on the design and optimization strategies of graphene surfaces, in particular the synthesis of graphene composites and the assessment of their performance potential towards their use in resistive gas sensors. Functionalization aspects are discussed, highlighting the properties of the graphene surface interaction with the target toxic gases, whilst also observing the shortcomings regarding response time, full recovery to baseline of the gas sensor, power consumption, and elevated temperatures. Modified graphene surfaces and their derivatives achieved by doping/functionalization have been displayed to overcome these issues by adopting metal oxide and/or heterostructured nanohybrids in effective synthetic routes towards sensitive and selective gas sensing. The synergistic effect of the graphene–metal oxide combination and proposed mechanisms have indicated that there are still more investigations to be done towards developing the next generation of graphene-based gas sensors. Therefore, it is pivotal for scientists to develop novel innovative materials which are reported at the lab level to compete with existing commercial technology in terms of good stability and the ability to operate for long times without any need for re-calibration.

## Figures and Tables

**Table 1:** Summary of short-term and long-term exposures to air pollutants shown by their mean concentrations and average exposures times following standards reported by WHO AQGs. Adapted from ref. [4].

Air Pollutant	Short-Term Exposure		Long-Term Exposure	
	Mean Concentration	Average Time	Mean Concentration	Average Time
O_3_	100 µg/m^3^	8 h	-	-
NO_2_	200 µg/m^3^	1 h	40 µg/m^3^	1 year
CO	100 mg/m^3^	15 min	60 mg/m^3^	30 min
			30 mg/m^3^	1 h
			10 mg/m^3^	8 h
SO_2_	500 µg/m^3^	10 min	20 µg/m^3^	24 h
PM_10_	50 µg/m^3^	24 h	20 µg/m^3^	1 year
PM_2.5_	25 µg/m^3^	24 h	10 µg/m^3^	1 year

## Data Availability

Not applicable.
